# O-13 - THE HEALTH IMPACTS FROM INDUSTRIAL SOURCES OF PFAS

**DOI:** 10.1093/pubmed/fdag046.013

**Published:** 2026-07-09

**Authors:** Miss Erin Eastman

**Affiliations:** UK Health Security Agency

## Abstract

**Background:**

Per-and poly fluoroalkyl substances (PFAS) are a large group of synthetic chemicals that have been produced since the 1940s for use in a broad range of consumer products and industries. They are extremely persistent and can accumulate over time in our bodies and the environment.

UKHSA's remit is to advise on the risks to public health from environmental exposure to chemicals, via soil, water and air. UKHSA recognises the growing concern with respect to potential risks to human health from PFAS and has been advising on several sites where PFAS is present.

**Objectives:**

In order to provide advice, UKHSA has undertaken a rapid review to answer: What are the potential adverse health effects associated with exposure to PFAS in populations living around industrial sites and manufacturing sites producing PFAS chemicals and products?

**Methods:**

The Cochrane Rapid Review guidance was followed, a robust search strategy used to identify papers on OVID Medline and OVID Embase. Duplicate screening undertaken for 10% of papers, with the 4 reviewers reviewing the remainder and discussing any disagreements.

Critical appraisal was undertaken utilising a bespoke framework which combines elements based on the guidance of evidence synthesis of epidemiological studies from the Synthesis and Integration of Epidemiological and Toxicological Evidence Subgroup, along with a scoring system developed by the US Environmental Protection Agency US EPA) in their PFAS Integrated Risk Information System (IRIS) assessments.

Evidence will be synthesised by health outcome, in line with NICE categorisations. Where there are any gaps/ uncertainties the International Classification of Disease (ICD-10), a global coding system by the World Health Organization will be referred to. Judgements on associations with health outcomes will be informed by the US EPA PFAS IRIS strength of evidence framework

**Results and Conclusions:**

The results and conclusions of the review are being finalised and will be published on the UK government's website in due course.

